# Subparalyzing Doses of Rocuronium Reduce Muscular Endurance without Detectable Effect on Single Twitch Height in Awake Subjects

**DOI:** 10.1155/2019/2897406

**Published:** 2019-05-02

**Authors:** Jan Gelberg, Peter Bentzer, David Grubb

**Affiliations:** ^1^Lund University, Skåne University Hospital, Department of Clinical Sciences, 221 85 Lund, Sweden; ^2^Lund University, Helsingborg Hospital, Department of Clinical Sciences, 221 85 Lund, Sweden

## Abstract

**Purpose:**

To test the hypothesis that a low-dose rocuronium acts mainly by means of reducing muscular endurance rather than by reducing momentary force.

**Methods:**

In a randomized placebo-controlled double-blinded study, eight healthy volunteers were studied in two sets of experiments. In the first set, the subjects made a sustained maximum effort with the dominant hand for 80 seconds while squeezing an electronic handgrip dynamometer at three minutes after intravenous administration of placebo, 0.04 or 0.08 mg/kg rocuronium. Handgrip force at initiation of testing (maximum handgrip force) and after 60 seconds was evaluated. In the second set, the ulnar nerve of the subjects was electrically stimulated every tenth second for at least 10 and a maximum of 30 minutes following the administration of placebo and 0.08 mg/kg rocuronium. Single twitch height of the adductor pollicis muscle was recorded.

**Results:**

There was no significant difference in the effect on maximum handgrip force at time 0 between the three different doses of rocuronium. As compared with placebo, handgrip force after 0.08 mg/kg rocuronium was reduced to approximately a third at 60 seconds (214 N (120–278) vs. 69 (30–166); *p*=0.008), whereas only a slight reduction was seen after 0.04 mg/kg (187 (124–256); *p*=0.016). Based on these results, the sustained handgrip force after 0.2 mg/kg at 60 seconds was calculated to be 1.27% (95% CI [0.40, 4.03]) of the maximum force of placebo. No effect on single twitch height after 0.08 mg/kg rocuronium at four minutes after drug administration could be detected.

**Conclusions:**

Subparalyzing doses of rocuronium show a distinct effect on muscular endurance as opposed to momentary force. The findings support the hypothesis that low doses of rocuronium act mainly by reducing muscular endurance, thereby facilitating, for example, tracheal intubation.

## 1. Introduction

There are situations when patients have to be intubated for short but maybe painful procedures requiring controlled ventilation. In such cases, the ideal anesthesia consists of a combination of a short-acting hypnotic, opioid, and muscle relaxant. One disadvantage with the standard intubating dose of rocuronium (0.6 mg/kg), equivalent to twice the 95% effective dose (ED_95_), is the time needed for recovery from muscle relaxation. For example, the spontaneous recovery time in infants given 0.6 mg/kg rocuronium to a train-of-four ratio (TOF ratio) > 0.7 was 64–95 min in one study [1]. To achieve excellent conditions for intubation without using muscle relaxants, generous doses of propofol and an opioid, remifentanil, or alfentanil are needed [2–4] which in turn delays awakening and risks compromising the hemodynamic stability of the patient. Furthermore, intubation without using muscle relaxants is associated with an increased risk of laryngeal trauma [5, 6].

Adding a low-dose rocuronium (0.1–0.3 mg/kg) to modest doses of propofol and remifentanil has in some studies appeared to be a method for successful intubation [3, 7, 8]. However, the mechanism of action of a low-dose rocuronium is not clear since it obviously does not rely on complete muscle paralysis. We hypothesized that a low-dose rocuronium primarily acts by reducing muscular endurance rather than momentary force, which is measured by peripheral nerve stimulation. The primary objective of this study in awake subjects was to test this hypothesis by evaluating the effect of subparalyzing doses of rocuronium on muscular endurance and momentary force, respectively. The ability to sustain handgrip force over time was used as a novel method to specifically assess muscular endurance. The procured data were used to simulate the effect on muscular endurance after a clinically relevant dose of 0.2 mg/kg rocuronium. Momentary force of the adductor pollicis muscle was assessed by ulnar nerve stimulation, the routine clinical practice.

## 2. Materials and Methods

After approval by the Ethical Review Board of Lund University (ref: 740/2005) and the Swedish Medical Products Agency (EudraCT ref: 2005-00522-31) and after written informed consent, eight healthy subjects were studied.

### 2.1. Preparations and Measurements

All subjects visited the laboratory on three different days. Day 1 was used only to get acquainted with the equipment.

On days 2 and 3, the subjects were placed in a supine position and blindfolded, not to reveal any ptosis to the observer. The subjects were breathing room air, and hemoglobin oxygen saturation was continuously measured from a finger probe. An intravenous catheter was placed in a cubital vein in the nondominant arm.

Syringes containing rocuronium (Esmeron®, Organon AB, Gothenburg, Sweden) at a dose of 0.04 mg/kg and 0.08 mg/kg, diluted with 0.9% sodium chloride to a volume of 10 ml or 10 ml of 0.9% sodium chloride (placebo), were prepared by an assistant nurse, not otherwise involved in the study.

At day 2, the subjects were asked to make a sustained maximum effort with the dominant hand for 80 seconds while squeezing an electronic handgrip dynamometer (GRIPPIT, AB Detektor, Gothenburg, Sweden) three minutes after administration of placebo, rocuronium 0.04 mg/kg, or rocuronium 0.08 mg/kg. This time interval was based on a reported time to max. effect of 2.3–3 minutes after rocuronium doses of 0.15–0.25 mg/kg [9, 10]. The subjects were continuously encouraged by the observer to maintain maximum strength. The device was automatically calibrated with every setup.

At day 3, two measurements with electric stimulations of the ulnar nerve using surface electrodes (Soft E-Kendall, Tyco Healthcare, Mansfield, MA, USA) were performed after injection of placebo or rocuronium 0.08 mg/kg. The neuromuscular function of the adductor pollicis muscle was monitored with TOF-Watch® SX (Organon AB, Gothenburg, Sweden). Single twitch stimulations were started with a frequency of 0.1 Hz, a duration of 200 *µ*s, and a current of 5 mA. The current was then stepwise increased until the first muscle twitch appeared. Thereafter, the current was further increased with 10 mA. Calibration was performed to set the existing twitch height to 100% (baseline). After calibration, stimulation was continued for five minutes before injection of placebo and rocuronium. After injection, stimulations continued until returning to more than 90% of baseline. The stimulations lasted for at least 10 minutes but not longer than 30 minutes after the injection.

The order between injections on the study days was randomized and blinded to both the observer and subject. Time between different measurements on both days was at least 90 minutes to allow for complete recovery of muscular strength. On days 2 and 3, the subjects had been fasting for at least six hours before drug administration, and there was full preparedness to take care of any untoward effect of nondepolarizing muscle blocking agents (NMBAs).

### 2.2. Data Analysis

Data from the handgrip force tests were acquired from the dynamometer by a software program (GrippitDA, AB Detektor, Gothenburg, Sweden) with a sampling rate of 10 Hz. The handgrip force of each subject at every tenth second was calculated as the mean from the ten data points obtained during 0.5 second before to 0.5 second after every tenth second. For construction of curve profiles over time, the maximum force for each individual reached within the first three seconds during the placebo measurement, was used as baseline.

Data from the single twitch stimulations were collected from the TOF-Watch® SX Monitor, version 2.2.INT (Organon Ltd., Dublin, Ireland). The evoked muscle response of each subject at every minute was calculated as the mean from the seven data points obtained during 30 seconds before to 30 seconds after every minute.

### 2.3. Statistics

Because this was considered to be a pilot study, no power analysis was performed. Data are presented using median (range) if not otherwise stated. The significance level was set to 5%, and no adjustments for multiple comparisons were done due to the explorative nature of the study.

Statistical comparisons between groups of handgrip force (N) and twitch height (%) were made using the Wilcoxon signed-rank test. Comparisons were made at 4 minutes after drug injection, which corresponds to handgrip force at 60 sec.

To estimate the effect of the different dose levels on handgrip force over time, a generalized linear mixed model having logarithmic % force as outcome and dose and time as predictors was used. The dose level was set as a categorical variable, and an interaction term between dose and time was included in the model to make effect comparisons between dose levels possible. As the decrease from time of max. force until 60 sec was of interest, the model included measurements from 3 until 60 seconds.

To predict handgrip force after a hypothetical 0.2 mg/kg dose of rocuronium at every second from 3 until 60 seconds, the same model as above was used, but the dose level was set as a continuous variable to make extrapolations possible. As there was no difference between placebo and 0.04 mg/kg, only dose levels 0.04 and 0.08 kg/mg were included in the model. For each second, a predicted value for handgrip force with associated confidence interval was calculated. SAS 9.4 and GraphPad PRISM version 7.0d and GraphPad software were used for the analyses.

## 3. Results

Eight healthy subjects, four males and four females, with an age of 22 years (21–29) and a BMI of 21.7 kg/m^2^ (20.3–24.7) were studied. None of the volunteers had any family history of neurological disease, and the only ongoing medication was oral contraceptives. There were no untoward events during the testing. Hemoglobin oxygen saturation remained stable during the observation period with the exception of one patient. In this patient, hemoglobin oxygen saturation decreased from 100 to 97% after administration of rocuronium at a dose of 0.08 mg/kg.

The maximum handgrip force was similar in the placebo and rocuronium 0.04 mg/kg group (360 N (198–483) vs. 352 (257–405); *p*=0.945), and a trend of a lower force was observed in the rocuronium 0.08 mg/kg group compared with placebo (317 N (199–364); *p*=0.055) (Table 1). Even though the subjects tried to maintain maximum force, it gradually decreased to 214 N (120–278) in the placebo group and to slightly lower in the rocuronium 0.04 mg/kg group (187 N (124–256); *p*=0.016) at 60 s. In the rocuronium 0.08 mg/kg group, the sustained grip force decreased to approximately one-third compared with placebo (69 N (30–166); *p*=0.008) (Table 1).

**Figure 1:**
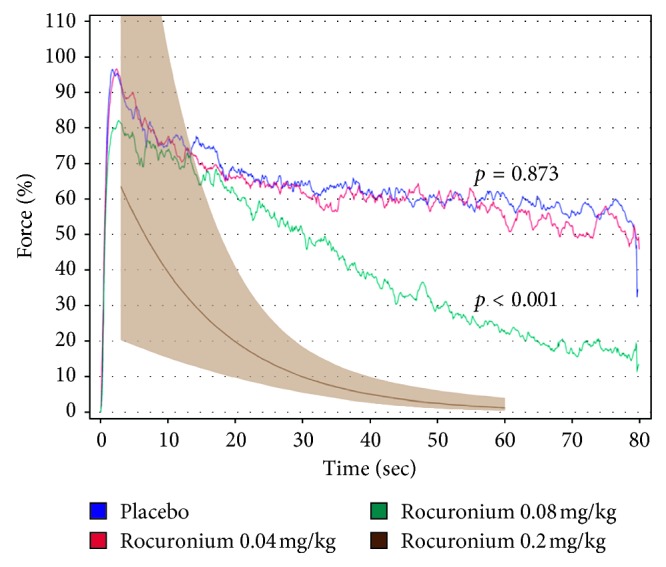
Average handgrip force over time from eight subjects after placebo, and rocuronium 0.04 mg/kg and 0.08 mg/kg was given. Subjects started to squeeze the dynamometer three minutes after placebo/rocuronium was given. A predicted curve between three minutes and 60 seconds with 95% confidence interval after a hypothetical dose of rocuronium 0.2 mg/kg is inserted. *P* values denote comparisons of average slope with placebo. The placebo curve does not reach 100% because subjects did not reach maximum at the exact same time point (see text for details).

The decrease over time was larger in the rocuronium 0.08 mg/kg group compared with placebo (2.45% reduction each sec, 95% CI [2.00, 2.90] vs. 0.81 [0.33, 1.29]; *p* < 0.001) but not in the 0.04 mg/kg group (0.76 [0.28, 1.24]; *p*=0.873), indicating a reduced ability to sustain muscular force over time in the 0.08 mg/kg group (Figure 1).

Modelling generated a predicted muscular force of 1.27%, 95% CI [0.40, 4.03] at 60 s after a hypothetical rocuronium dose of 0.2 mg/kg (Figure 1).

Twitch height at the time of injection was similar in the placebo and in the rocuronium group (106% (95–114) vs. 108% (92–132); *p*=0.641). Four minutes later, there was still no significant difference between groups (105% (94–119) vs. 100 (85–106); *p*=0.055) (Table 2). The currents used for single twitch stimulation were 22.5 (19–26) mA in the placebo and 23.5 (20–27) mA in the rocuronium group (*p*=0.999). One subject in the placebo and two subjects in the rocuronium group did not return to more than 90% of baseline.

## 4. Discussion

The main finding of the present study is that rocuronium at a dose of 0.08 mg/kg reduces the handgrip strength at 60 seconds to approximately one-third after placebo whereas the effect was barely discernible after 0.04 mg/kg rocuronium. No effect on maximum force at start of testing of handgrip strength was detected. The dose-dependent effect of rocuronium at these doses enabled the calculation of the effect corresponding to a therapeutic dose given for anesthetic purposes. Thus, 0.2 mg/kg rocuronium (67% of ED_95_) was predicted to decrease handgrip strength to approximately 1% at 60 seconds compared to placebo.

The ED_95_ of an NMBA denotes the dose required to reduce twitch height by 95% which corresponds to 0.3 mg/kg for rocuronium [11]. While the standard intubating dose is 0.6 mg/kg, lower doses (0.1–0.3 mg/kg) have been suggested to avoid the inherent long recovery time of 0.6 mg/kg rocuronium [3, 12–14]. We chose to predict the effect of 0.2 mg/kg rocuronium since the recovery time to TOF >0.9 of that dose has been found to be 23 minutes in one study [15], which ought to be acceptable even for a short surgical procedure. Due to individual variability of sensitivity to NMBAs, it is difficult to foresee which NMBA doses are safe and at the same time, produce measurable effects. Previous studies on awake subjects have shown variable effects of different NMBAs on various parameters in the dose range of 13–35% of ED_95_ [16–20]. Therefore, rocuronium doses of 13 and 27% of ED_95_ were used in this study.

The reduction of maximum grip strength after NMBA has previously been studied [20, 21]. Isono et al. found a 12% reduction after 0.02 mg pancuronium (29% of ED_95_), and Kopman et al. found a 43% reduction in maximum strength after titrating mivacurium to achieve a TOF of 0.7. However, to our knowledge, the effect on sustained handgrip strength of NMBA measured with an electronic dynamometer has not been studied before. Electronic as compared with hydraulic dynamometers provides additional information such as average grip strength over a set time period, less variability within subjects, and a higher sensitivity to abnormality [22].

The finding that subparalyzing doses of rocuronium reduced the ability to maintain the muscle force in the hand may explain how a low NMBA dose can facilitate intubation, namely, by reducing the ability to maintain the adduction force of the larynx. However, for several reasons, it is difficult to generalize muscular effects in the hand to laryngeal muscles. In general, the neuromuscular blockade at the laryngeal muscles is less intense and with a more rapid onset and offset compared to the adductor pollicis muscle [9, 23]. This means that, to achieve the same effect on the laryngeal muscles as in the hand, a larger dose has to be given. However, these results do not necessarily apply to muscular endurance. Laryngeal muscles maybe exquisitely susceptible to the “fatigability” effect of NMBA considering that the intrinsic muscles of the larynx consist mostly of fast-twitch type II fibers in contrast to the adductor pollicis muscle [24] and that type II fibers show less endurance to work. Certainly, this reasoning needs to be corroborated in other studies.

While the effect of 0.08 mg/kg rocuronium (27% of ED_95_) on sustained muscle force was obvious, no effect on muscle function as measured by twitch height of the thumb could be detected. Previous studies on twitch height by ulnar nerve stimulation after subparalyzing doses of NMBA have shown some effect. Aziz et al. [14] found a decrease in the mean TOF-ratio to 0.89 after 0.06 mg/kg rocuronium (20% of ED_95_), and Howardy-Hansen et al. [17] found a decrease of the median TOF ratio from 0.96 to 0.89 after 0.015 mg/kg pancuronium (21% of ED_95_). Although these results suggest TOF measurements to be a more sensitive method, 0.1 Hz single twitch measurement was chosen because during the onset of NMBAs, which represents the time phase of the intubation maneuver, the decrease of twitch height is faster than the development of fade in TOF measurements [25–29].

A frequency of 0.1 Hz and a stabilization period of five minutes before drug injection were applied to avoid the gradual decrease seen in evoked response with frequencies ≥0.15 Hz [30] and drift in twitch height which is described during the first two to three minutes after calibration [31].

Perhaps, the most plausible explanation to the lack of effect on single twitch height was the use of relatively low median currents, 22.5 and 23.5 mA in each group. Although TOF monitoring has been found to be stable in the range of 20–30 mA in previous studies, amperage was at the same time recommended to be set 10–25 mA above threshold current [32–35]. Despite this uncertainty, it felt important to keep the current as low as possible because of reported discomfort at current intensities around 50 mA and sometimes even at lower currents [32, 36, 37].

Three subjects, including one in the placebo group, never returned to above 90% of baseline twitch height. This fact, beside the absence of any significant NMBA effect, implies that the single twitch model used in the present study needs refinement.

Strengths of this study include the randomized double-blinded design whereas the most obvious study limitation is that the method used to assess muscular endurance, handgrip strength, is not possible to apply in a clinical setting of unconscious patients. The present study, however, was merely designed to test the existence of an alternative physiological effect of low doses of rocuronium. In light of this study, future NMBA research might consider the existence of effects that are not investigated by very brief nerve stimulations. Other limitations of the study include the low number of subjects which increases the uncertainty of the results, especially the calculated data, and the likelihood that the study is underpowered to detect differences in, for example, twitch height between groups. Furthermore, the study population may not be a representative of sick, old, and very young patient groups.

## 5. Conclusion

Subparalyzing doses of rocuronium showed a distinct effect on sustained handgrip strength as a measure of muscular endurance. There was no corresponding effect on twitch height and measuring momentary force. The findings support the hypothesis that a low dose of rocuronium acts mainly by reducing muscular endurance, thereby facilitating, for example, tracheal intubation.

## Figures and Tables

**Table 1:** Effect of placebo and different doses of rocuronium on sustained force (median (range)).

Time (s)	Placebo (*N*)	Rocuronium 0.04 mg/kg (*N*)	Rocuronium 0.08 mg/kg (*N*)
Max. force	360 (198–483)	352 (257–405) n.s.	317 (199–364) n.s.
10	260 (154–363)	256 (210–316)	240 (200–342)
20	246 (153–298)	222 (141–314)	194 (168–285)
30	234 (126–282)	222 (157–257)	149 (125–247)
40	222 (129–310)	217 (145–269)	126 (80–215)
50	203 (138–290)	216 (155–248)	92 (54–159)
60	214 (120–278)	187 (124–256)^*∗*^	69 (30–166)^*∗∗*^
70	208 (120–254)	175 (127–218)	47 (33–127)

n.s., not significant; ^*∗*^*p* < 0.05; ^*∗∗*^*p* < 0.01 as compared with placebo. The Wilcoxon signed-rank test was used.

**Table 2:** Effect of placebo and rocuronium 0.08 mg/kg on twitch height (median (range)).

Time (min)	Placebo (%)	Rocuronium 0.08 mg/kg (%)
0	106 (95–114)	108 (92–132) n.s.
1	105 (94–113)	103(92–125)
2	107 (96–114)	102 (89–115)
3	107 (97–115)	102 (86–111)
4	105 (94–119)	100 (85–106) n.s.
5	106 (92–121)	100 (85–103)

n.s., not significant compared with placebo. The Wilcoxon signed-rank test was used.

## Data Availability

The data used to support the findings of this study are available from the corresponding author upon request.
